# Supplementary material for: Arrayed Imaging Reflectometry monitoring of anti-viral antibody production throughout vaccination and breakthrough Covid-19
Source: PLoS One. 2023 Feb 7;18(2):e0277846. doi: 10.1371/journal.pone.0277846 (PMC9904502; doi:10.1371/journal.pone.0277846)
Supplement: S1 Table — (DOCX) [file pone.0277846.s001.docx]

| **Array Protein** | **LOD (Å)** |
| --- | --- |
| RSV A G Protein | 1.9 |
| RSV B G Protein | 2.6 |
| Influenza A Beijing H1N1 | 4.7 |
| Influenza A California H1N1 04-2009 | 1.9 |
| Influenza A California H1N1 07-2009 | 2.3 |
| Influenza A Guangdong-Maonan H1N1 | 8.6 |
| Influenza A Hong Kong 2014 | 2.0 |
| Influenza A Hong Kong H3N2 2019 | 7.1 |
| Influenza A Shanghai H7N9 | 9.1 |
| Influenza A Switzerland H3N2 | 2.2 |
| Influenza A Texas H3N2 | 1.7 |
| Influenza A Vietnam H5N1 | 2.2 |
| Influenza A Wisconsin H3N2 | 6.9 |
| Influenza B Brisbane | 3.0 |
| Influenza B Florida 2006 | 5.3 |
| Influenza B Malaysia | 4.0 |
| Influenza B Massachusetts | 3.3 |
| Influenza B Phuket | 3.9 |
| Influenza B Washington 2019 | 5.7 |
| CoV-229E Spike (S1+S2) | 8.3 |
| CoV-HKU1 S1 | 3.4 |
| CoV-NL63 S1 | 2.5 |
| CoV-OC43 Spike (S1+S2) | 4.7 |
| MERS-CoV Nucleocapsid | 2.0 |
| MERS-CoV RBD | 3.6 |
| MERS-CoV S1 | 2.6 |
| SARS-CoV RBD | 4.2 |
| SARS-CoV S1 | 2.3 |
| SARS2-CoV-2 Nucleocapsid | 7.7 |
| SARS-CoV-2 RBD | 3.1 |
| SARS-CoV-2 S1 | 4.3 |
| SARS-CoV-2 Spike (S1+S2) | 2.3 |
| SARS-CoV-2 S2 | 4.3 |
| SARS-CoV-2 S1 D614G mutation | 6.0 |

S1 Table**.** Limits of detection in thickness for each protein probe on the array used for vaccine study.
