# Supplementary material for: Arrayed Imaging Reflectometry monitoring of anti-viral antibody production throughout vaccination and breakthrough Covid-19
Source: PLoS One. 2023 Feb 7;18(2):e0277846. doi: 10.1371/journal.pone.0277846 (PMC9904502; doi:10.1371/journal.pone.0277846)
Supplement: S2 Table — (DOCX) [file pone.0277846.s002.docx]

| **Influenza strain** | **Full length name** |
| --- | --- |
| Influenza A Beijing H1N1 | A/Beijing/22808/2009 |
| Influenza A California H1N1 04-2009 | A/California/04/2009 |
| Influenza A California H1N1 07-2009 | A/California/07/2009 |
| Influenza A Guangdong-Maonan H1N1 | A/Guangdong-Maonan/SWL1536/2019 |
| Influenza A Hong Kong 2014 | A/Hong Kong/4801/2014 |
| Influenza A Hong Kong H3N2 2019 | A/Hong Kong/2671/2019 |
| Influenza A Shanghai H7N9 | A/Shanghai/1/2013 |
| Influenza A Switzerland H3N2 | A/Switzerland/9715293/2013 |
| Influenza A Texas H3N2 | A/Texas/50/2012 |
| Influenza A Vietnam H5N1 | A/VietNam/1203/2004 |
| Influenza A Wisconsin H3N2 | A/Wisconsin/67/2005 |
| Influenza B Brisbane | B/Brisbane/60/2008 |
| Influenza B Florida 2006 | B/Florida/4/2006 |
| Influenza B Malaysia | B/Malaysia/2506/2004 |
| Influenza B Massachusetts | B/Massachusetts/03/2010 |
| Influenza B Phuket | B/PHUKET/3073/2013 |
| Influenza B Washington 2019 | B/Washington/02/2019 |

S5 Table. Full length names of Influenza strains used in the Ziva arrays.
