# Supplementary material for: Arrayed Imaging Reflectometry monitoring of anti-viral antibody production throughout vaccination and breakthrough Covid-19
Source: PLoS One. 2023 Feb 7;18(2):e0277846. doi: 10.1371/journal.pone.0277846 (PMC9904502; doi:10.1371/journal.pone.0277846)
Supplement: S1 Fig — (DOCX) [file pone.0277846.s003.docx]

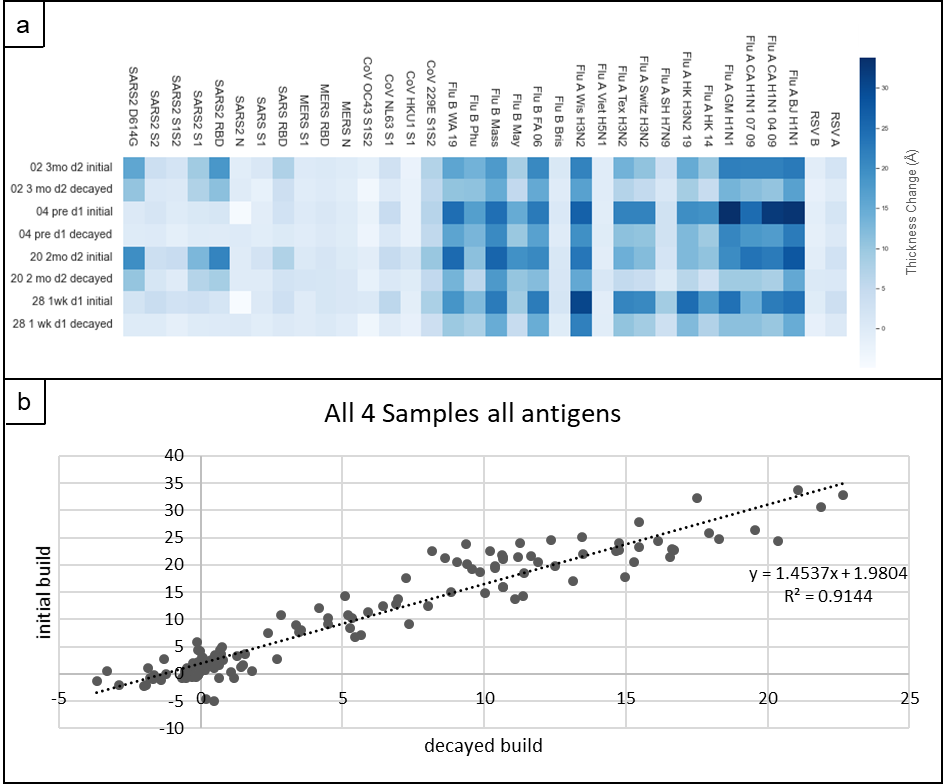


S2 Figure. Comparison of the four samples run on both the newly opened 34-plex ARVA array (initial) and the same array after 6 months of the array stored in the 4°C refrigerator (decayed). The samples were different aliquots stored in the -80°C freezer. The thickness change is clearly muted on the decayed array (a). All antigen thickness changes (build) for all samples were plotted to find a relationship between build on the initial array and build on the decayed array. Linear regression demonstrated a strong relationship (R^2^=0.91), and the equation was used to adjust all of the builds run on the 6-month-old array back up to initial levels (b).
