# Supplementary material for: Arrayed Imaging Reflectometry monitoring of anti-viral antibody production throughout vaccination and breakthrough Covid-19
Source: PLoS One. 2023 Feb 7;18(2):e0277846. doi: 10.1371/journal.pone.0277846 (PMC9904502; doi:10.1371/journal.pone.0277846)
Supplement: S2 Fig — (DOCX) [file pone.0277846.s004.docx]

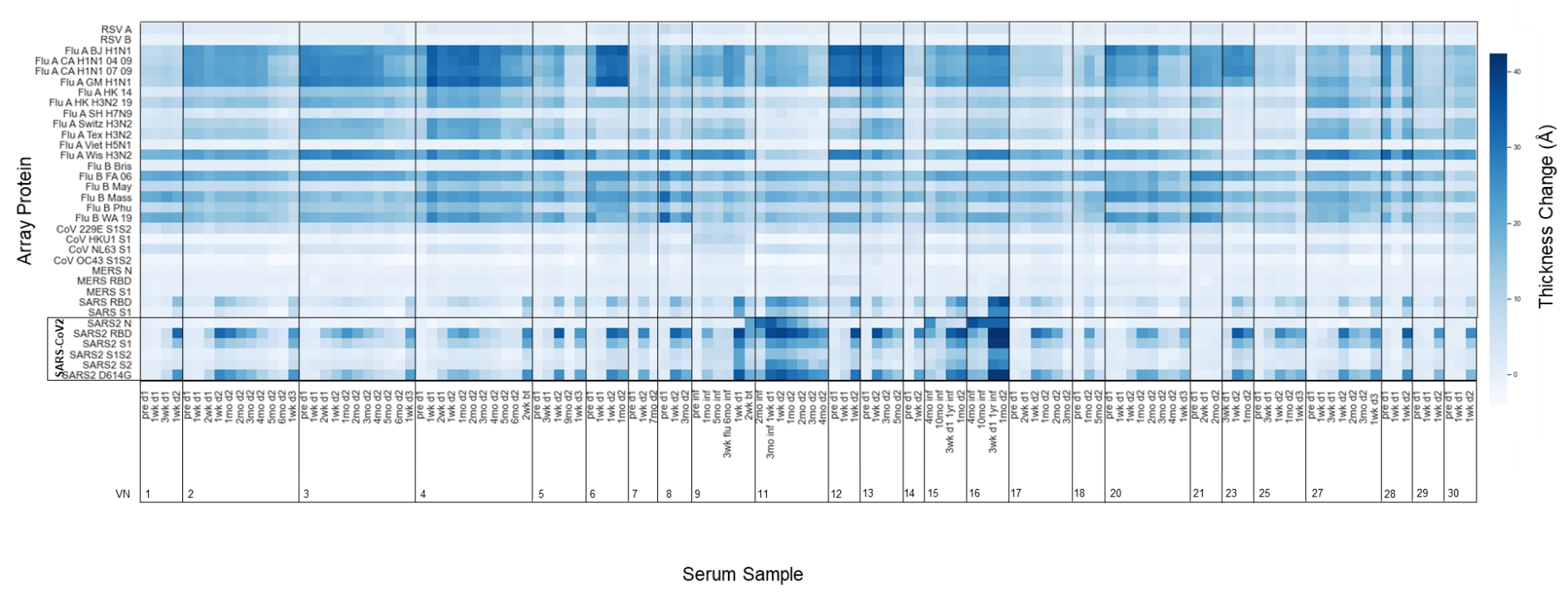


S3 Figure. All unadjusted thickness changes for the longitudinal vaccine samples shown in figure 4 of the main text.
