# Supplementary material for: Arrayed Imaging Reflectometry monitoring of anti-viral antibody production throughout vaccination and breakthrough Covid-19
Source: PLoS One. 2023 Feb 7;18(2):e0277846. doi: 10.1371/journal.pone.0277846 (PMC9904502; doi:10.1371/journal.pone.0277846)
Supplement: S3 Fig — Box plots of thickness (Å) due to antibody binding in Covid-19 convalescent serum vs serum from individuals uninfected with Covid-19 on the 16-plex ARVA array. (DOCX) [file pone.0277846.s005.docx]

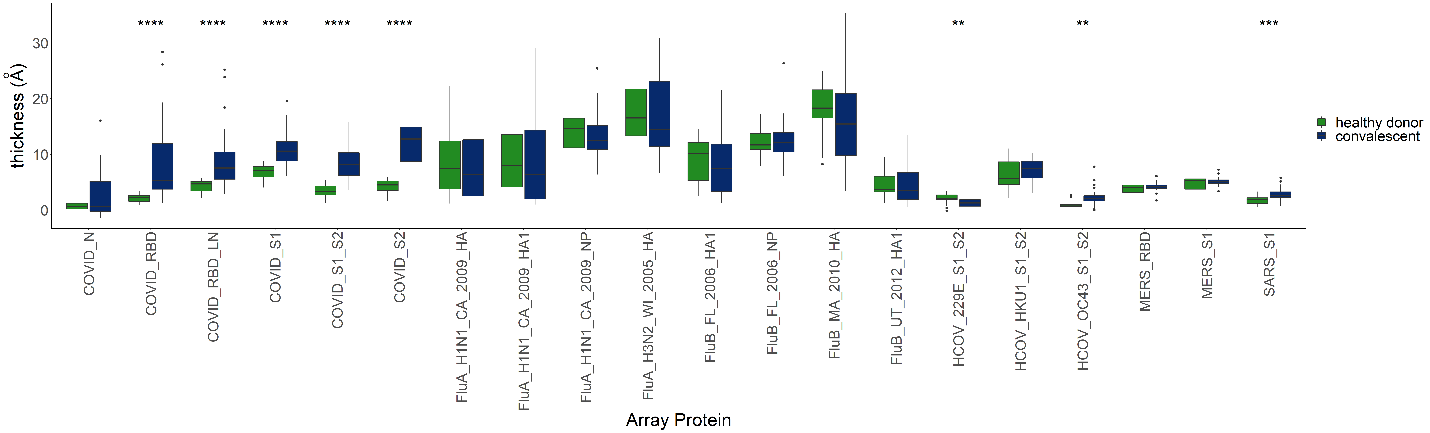


S4 Figure. Box plots of thickness (Å) due to antibody binding in Covid-19 convalescent serum vs serum from individuals uninfected with Covid-19 on the 16-plex ARVA array. Boxplots display the median value and data points between the 25^th^ and 75^th^ percentile (boxed), the minimum and maximum data points (tails) along with potential outliers (dots). Significance determined by unpaired, two tailed t-test assuming unequal variance, ****p<0.0001***p<0.001, **p<0.01, *p<0.05.
