# Supplementary material for: Arrayed Imaging Reflectometry monitoring of anti-viral antibody production throughout vaccination and breakthrough Covid-19
Source: PLoS One. 2023 Feb 7;18(2):e0277846. doi: 10.1371/journal.pone.0277846 (PMC9904502; doi:10.1371/journal.pone.0277846)
Supplement: S4 Fig — Box plots of thickness (Å) due to antibody binding after incubation in serum from individuals prior to vaccination (no previous infection with Covid-19) and 1–2 weeks after second dose of either a Pfizer/BioNTech (BNT162b2) or Moderna (MRNA-1273) vaccine. (DOCX) [file pone.0277846.s006.docx]

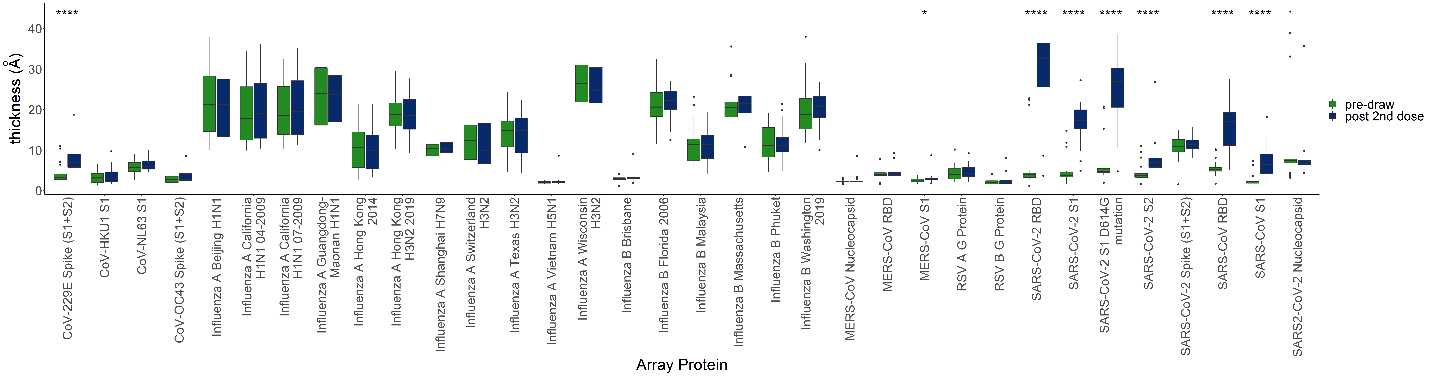


S6 Figure. Box plots of thickness (Å) due to antibody binding after incubation in serum from individuals prior to vaccination (no previous infection with Covid-19) and 1-2 weeks after second dose of either a Pfizer/BioNTech (BNT162b2) or Moderna (MRNA-1273) vaccine. Boxplots display the median value and data points between the 25^th^ and 75^th^ percentile (boxed), the minimum and maximum data points (tails) along with potential outliers (dots). Significance determined by paired, two tailed t-test assuming unequal variance, ****p<0.0001***p<0.001, **p<0.01, *p<0.05.
